# Genetic interactions matter more in less-optimal environments: a Focused Review of “Phenotype uniformity in combined-stress environments has a different genetic architecture than in single-stress treatments” (Makumburage and Stapleton, 2011)

**DOI:** 10.3389/fpls.2014.00384

**Published:** 2014-08-11

**Authors:** Dustin A. Landers, Ann E. Stapleton

**Affiliations:** Department of Biology and Marine Biology, University of North Carolina WilmingtonWilmington, NC, USA

**Keywords:** QTL, genotype-environment interaction, modifier, uniformity, variance heterogeneity, combined stress effects, abiotic stress, crop

## Abstract

An increase in the distribution of data points indicates the presence of genetic or environmental modifiers. Mapping of the genetic control of the spread of points, the uniformity, allows us to allocate genetic difference in point distribution to adjacent, cis effects or to independently segregating, trans genetic effects. Our genetic architecture-mapping experiment elucidated the “environmental context specificity” of modifiers, the number and effect size of positive and negative alleles important for uniformity in single and combined stress, and the extent of additivity in estimated allele effects in combined stress environments. We found no alleles for low uniformity in combined stress treatments in the maize mapping population we examined. The major advances in this research area since early 2011 have been in improved methods for modeling of distributions and means and detection of important loci. Double hierarchical general linear models and, more recently, a likelihood ratio formulation have been developed to better model and estimate the genetic and environmental effects in populations. These new methods have been applied to real data sets by the method authors and we now encourage additional development of the software and wider application of the methods. We also propose that simulations of genetic regulatory network models to examine differences in uniformity and systematic exploration of models using shared simulations across communities of researchers would be constructive avenues for developing further insight into the genetic mechanisms of variation control.

## Introduction

**Figure 1:**
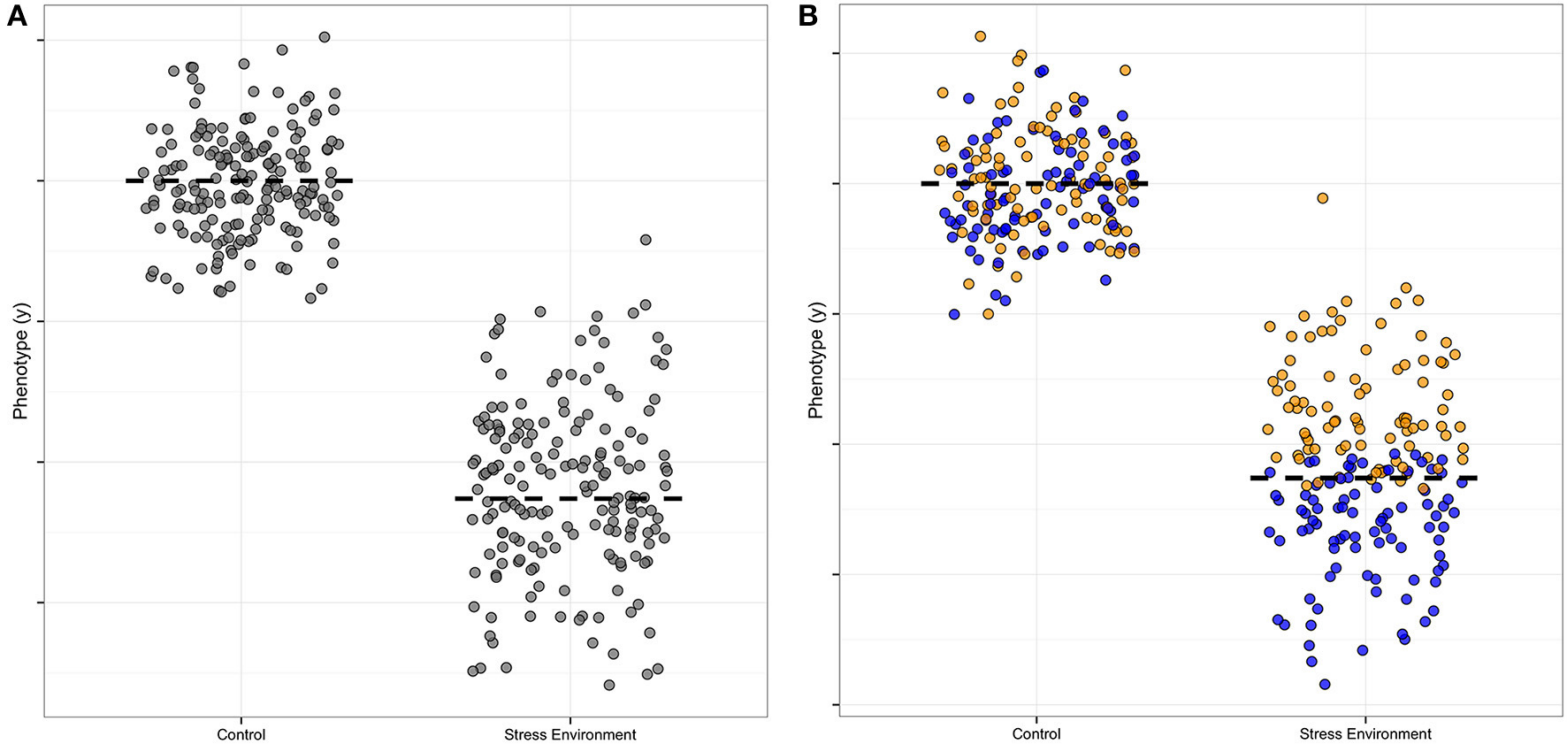
**Hypothetical example of a modifier effect on a plasticity allele**. This illustration is formulated like Figure 1 in Ronnegard and Valdar (2012), except that our focus is on environmental factors rather than allele interactions. The y axis is the measured amount of a phenotype, and the x axis indicates the effect of a single allele of a plastic, environmentally-sensitive locus. **(A)** The stress-sensitivity is apparent in the difference in the mean (dashed line) contribution to the phenotype of the allele, with high phenotype effect in the normal control setting and low contribution in the stress environment. The spread of the points also differs for this allele, with a tight clustering of points around the mean in the control and a wider spread in the stress environment. **(B)** A modifier allele is illustrated by color-coding the phenotype effect estimates. In this example the modifier confers a higher mean (yellow points) and the plasticity allele retains its sensitivity to environmental stress (blue points). The mean difference is still visible in this example, though with the modifier identified the mean plasticity of the focus allele would be even larger than originally estimated from part **(A)**.

There is useful information in the distribution of data as well as the mean (Cleasby and Nakagawa, 2011; Geiler-Samerotte et al., 2013); genetic analysis of distributions can be especially informative (Hill and Mulder, 2010; Ronnegard and Valdar, 2012). Specifically, an increased spread of measured allele effects indicates the presence of a **modifier**, and is thus a clue to biological mechanisms. In Figure 1, we illustrate this point by showing the increased spread around the average in one symbol shape (Figure 1A, where the normal environment has points clustered around the mean where the stress environment has points spread broadly up and down from the mean), and then illustrate how the presence of a modifier that increases the growth trait under stress could be visualized, using blue filled symbols as compared to the yellow unfilled symbols (Figure 1B, compare normal to stressed effect). The color-coding thus represents the additional “dimension” when a modifier is present. In our hypothetical Figure 1 example, the modifier ameliorates the stress effect of the allele, as without the blue modifier points' contribution to the mean, the average value for the trait of any organism carrying that particular allele would be even lower than the two-fold decrease shown in the Figure 1 example. Of course it is also possible to have equal means when modifiers are present, which would mean that the allele is not detectable as a separate genetic variant. This type of modifier masking, without a detectable mean effect, could thus contribute to false negatives in typical quantitative trait locus (**QTL**) analyses of **genetic architecture**.

KEY CONCEPT 1. Modifier allele or alleles that change the measured phenotype effect of another allele. This definition implies that the modifier effect is heritable and that the modifier allele effect is only measurable when the “receiving” genetic variant is present.

KEY CONCEPT 2. QTL (quantitative trait locus) a particular allelic difference between DNA molecules that is associated with a difference in a measured phenotype of the organisms. To do such an analysis, there must be variation in the genotype (SNPs, markers) *and* variation in the phenotype (trait, measured value of trait).

KEY CONCEPT 3. Genetic architecture list of the number of alleles and the pattern of allele effects in a genotype-phenotype mapping experiment. This can range from one or two large-effect alleles to many very small-effect alleles, or a mixture of these types. Most mapping populations only allow detection of relatively large effect SNPs (down to about 1% of the total variation of the measured trait); we assume that additional undetectable small-effect variants are present.

Our simple hypothetical example in Figure 1 includes both a modifier and an environmental difference. Environment-specific variants have been studied extensively (Lynch and Walsh, 1998), and are known as **plasticity** alleles. Analysis of these alleles, or of genotypes that include a variety of these alleles, differs depending on the goal of the analysis. For plant breeding, alleles that are conditionally neutral or are favorable across all environments are chosen (Lynch and Walsh, 1998). For a recent pictoral illustration of plasticity allele effect classifications, please see the review by El-Soda et al. (2014). In some breeding studies, alleles with effects that assist in classifying sets of target environments are included in trials (Windhausen et al., 2012). These standard genotypes can allow grouping of test sites into suitable target production environment predictors, and thus improve the efficiency of selective breeding programs. Information on important features across sets of environments can also be derived from crop and weather model parameters (Heslot et al., 2014) to better link gradient or factorial variables with chromosomal alleles.

KEY CONCEPT 4. Plasticity difference in genetic architecture in a comparison of environments. This term is normally used in comparisons of the same population, so that the genetic variation is held constant while the environment is varied.

Basic research on the evolutionary trajectories and specific interactions that underlie genetic architecture differences has incorporated environment-specific differences, though combinations of stress have rarely been examined. In this focused review we emphasize multiple-factor combinations as an intermediate between single-factor lab-scale experiments and large-scale environmental dissection or clustering, such as crop modeling and weather record covariate analyses. Multiple-stress effects are relevant to breeding for our growing population, as typical crop yields are substantially lower than yields under optimum conditions, and the limiting factors vary. This yield gap could theoretically be narrowed by breeding more tolerant genotypes (Tollenaar and Lee, 2002), though the interactions between stresses complicate selection. Typical crop environments are composed a mixture of different limiting factors, with variation across the growth season as well as season to season. There is relatively little information about combinations of limiting, stressful environmental factors (Sharma et al., 2013), which motivated us to map loci important for single stress and combined stress using a factorial approach. We focused specifically on the genetic architecture of **uniformity** by environment interactions, as modifiers are key in understanding mechanism, so that we can move toward more general predictability as well as prediction based on environment context-dependent genetic variants. We encourage researchers who are designing genetic architecture comparisons to consider experimental designs that allow fitting of these variance-control-detection models that will allow identification of new aspects of the genotype-phenotype map.

KEY CONCEPT 5. Uniformity spread of a group of measurements of experimental units such as individual plants; high uniformity indicates that most of the measurements in the replicates are close to the average, whereas low uniformity indicates that many points are far from the average of the replicates. The value of this number will scale with the value of the mean unless adjusted.

## Summary of the main results of our frontiers article (Makumburage and Stapleton, 2011)

The three key results of our mapping experiment include the “context specificity” of modifiers, comparison of the number and effect size (major QTL or minor QTL) of positive and negative alleles for uniformity in single and combined stress, and the extent of additivity in estimated allele effects in combined stress environments. To recap the first point, modifiers that map to the same allele as the mean effect (cis alleles) would be more likely to be transmitted together through the generations and thus not be dependent on the population context, as compared to trans alleles that could segregate independently. We found nine trans alleles for maize plant height trait uniformity, with these nine modifying loci spread over seven of the ten maize chromosomes. These nine loci are trans alleles, as there was no QTL at the same locus for mean amount of height. We detected only one coincident cis QTL (in other words, one locus that had a significant effect on both uniformity and amount of height). That single coincident QTL had different allele effect patterns across environments for height and uniformity, so it is not strictly cis in effect. Thus, the modifiers of plant height in this maize IBM94 **RIL** mapping population are appeared to primarily segregating independently from the loci that contribute directly to tall and short plants in our experiment.

KEY CONCEPT 6. RIL recombinant inbred line; these are experimental populations derived from the crossing of two inbred parents and subsequent inbreeding, so that cross-overs are now visible as “chunks” of the genome from each parent in each RIL. “Clones” of the same genotypes can be tested in many environments, and the lines have a known ancestral origin so that presence of a SNP can be modeled as independent from SNPs on other chromosomes.

Secondly, under single stress conditions there are stress-specific alleles that are high and low uniformity as compared to the population mean, but we only detected alleles important for uniformity decrease under combined stress. This pattern is different than the architecture of mean plant height, which has loci with both high and low allele effects under combined stress. In our hypothetical example (Figure 1B), the yellow-point modifier effect confers stress environment tolerance, and thus reduces the effect size of the blue allele under stress. This particular modifier interaction example was chosen as it illustrates a common modifier pattern in our data, with the stress modifier reducing the effect size of the allele at a locus. Thus, the stress-specific modifier would increase the spread of the points (decreasing the uniformity) as it conferred tolerance to the stress. All the uniformity alleles we found do indeed decrease uniformity, though we could not separate the effects of the modifier and the allele in the way we color-coded our hypothetical example, as we carried out separate Levene and mean analyses and compared them by map overlay. New methods for detecting modifier contributions are discussed in the next section of this review.

Finally, we found six loci that had predictable multiple-stress allele uniformity effects and three loci with surprising allele effects that could not be extrapolated from single stress effect estimates. One-third non-additive is probably an underestimate, as we first identified our QTL as having at least one significant genotype-environment interaction. After these G x E loci were identified we examine the allele effects post hoc. We did not fit models designed to test for modifier contributions as illustrated in our example (Figure 1B). Fitting of models designed to specifically detect effects jointly in combined and single stress and to also separate modifier contributions from mean effects would be useful in future analyses, as such more specific models for additive or multiplicative combinations might be expected to increase our ability to detect higher-order effects even in small data sets. We discuss this point in more detail in the “Areas for Future Work” section below.

## Review of related results since our frontiers publication

The major advance in this area since early 2011 has been the development of improved methods for modeling of distributions and means and detection of important loci. Double hierarchical general linear models and, more recently, a likelihood ratio formulation have been developed to better model and estimate the genetic and environmental effects in populations. There have also been applications of the models to new data sets and application of straightforward statistical models to well-understood biological data since our publication appeared.

Conventional statistical methods, while still popular, have some inherit pitfalls that have been addressed by more recent methods. Levene's test (the method used in the article that is the focus of this review) is obtained as the absolute differences between each observation and its group's mean or median. Generally, an F-statistic is used to make inferences about the trait's uniformity. This test is relatively robust to the distribution of the data points. Unfortunately, the test is unable to compare means and uniformity simultaneously and lacks the capacity to include covariates directly in the analysis.

In determining how much organisms' genetics control responses to environments, a novel statistical method was developed that used double hierarchical generalized linear models (DHGLM) to estimate the genetic variance of both macro- and micro-**environmental sensitivity** simultaneously (Ronnegard and Valdar, 2012). In this formulation, macro-environmental factors can be thought of as those that are known and can be measured while micro-environmental factors are unknown. The most recent published DHGLM analysis (Mulder et al., 2013b) combines two models: one model to estimate the genetic variance of macro-environmental sensitivity, expressed as the genetic variances in the intercept and slope of a reaction norm curve (where the reaction norm is the difference between two environments; if the difference is connected with a line the slope of the line can be used as the amount of the environmental difference), combined with a generalized linear model for the residual variance in order to estimate genetic variances in micro-environmental sensitivity, expressed as the differences in environmental variance. The implementation requires iteration between the two models and updating the weight matrices used in the iteratively weighted least squares estimation method until variance component estimates converge (i.e., estimates become relatively stable). The DHGLM method was shown to have generally low bias in most cases, with special attention to be paid to the relatively low bias present in the genetic parameters even when the estimated statistical model differed from the true genetic model. However, precision was usually not very high, especially in the estimation of the genetic variance of micro-environmental sensitivity (i.e., standard deviations across replicates were very high). This was especially true in designs with small families; the authors used an animal breeding model and recommended designs with at least 100 sire families each with at least 100 offspring each in order to ensure reasonably high precision.

KEY CONCEPT 7. Environmental sensitivity difference in measured phenotype value when the population is examined in different environments. The difference in environments can be loosely specified, such as difference in season or site. Alternatively, environments can be varied in a tightly specified way, as factorial experiments with all other aspects controlled.

In addition to the offspring size effect on precision, the inclusion of additional fixed effects in the models could further increase both the required family size and required offspring per family. However, the required offspring numbers for detecting genetic variance in macro-environmental sensitivity is generally lower than for differences in micro-environmental sensitivity and this expectation was observed in the authors' analysis. Further, the environmental parameter used for the reaction norm slope was assumed to be known and without error. In many cases, this might be fine, but there may be specific situations in which researchers may wish to consider environmental parameters estimated from the data. Unfortunately, environmental parameters that are estimated from the data were shown to seriously bias the estimations of genetic variance in macro-environmental sensitivity. Provided these limitations are addressed in the experimental design, the DHGLM method remains a very useful method to increase our understanding of the genetic variance of environmental sensitivity as well as provide us with tools for discriminating between these types of environmental sensitivity.

A more recently developed method (Cao et al., 2014) for detecting quantitative trait loci with **variance heterogeneity** (vQTL) is a likelihood ratio test developed to test differences in means and variances simultaneously (LRT_MV_). Derivatives of this test allow for single-purpose testing of variance heterogeneity (i.e., phenotype uniformity) and mean differences (notated LRT_V_ and LRT_M_ respectively). This omnibus method tests for these differences by comparing a full model with a null model that lacks certain properties with the purpose of evaluating the differences in likelihood. The full model requires dummy/indicator predictors for genotype (for example, for random population samples for the major allele homozygous, heterozygous, and minor allele homozygous classes) and a heterogeneous residual variance (a different residual variance for each allele type). It also permits the inclusion of a covariate matrix (e.g., sex, subpopulation structure principal components, etc.). The null model's specification depends on the purpose of the test, for which the authors outlined three. Specifically, in a scenario where differences in means and variances are to be tested (LRT_MV_), the null model simply excludes terms for both genotype and models the residual variance as homogenous (i.e., modeling all genotypes as having identical variance), effectively leaving a model with an intercept, covariates, and normal error term. Similarly, if the scenario only requires us to test the uniformity of the phenotype (LRT_V_), then the null model is identical to the full model, except again, the residual variance is modeled as homogenous. Finally, if the test required only the differences in means to be tested (LRT_M_), then the null model would be specified as having unique residual variances per genotype, but exclude dummy predictors for genotype.

KEY CONCEPT 8. Variance heterogeneity differences in the spread of points between different samples or factors in an experiment; this is similar to uniformity but with the addition of the comparison of more than one experimental unit to the meaning.

The purpose of the likelihood ratio tests (LRT_MV_, LRT_M_, and LRT_V_) is to compare the likelihood of both the full and null model given observed phenotypes and using that to draw conclusions about the presence of mean differences and/or variance heterogeneity. Under Wilks's theorem, the distribution of the likelihood ratio tests follows an approximate chi-squared distribution, permitting us to draw conclusions with significance testing. The LRT_V_, however, is closely related to Bartlett's test of equality of variance, which has shown to be sensitive to even slight violations of the normality assumption. Simulation studies of the LRT showed that LRT_MV_ and LRT_V_ (both including test of variance heterogeneity) indeed have inflated Type I errors, and thus the authors recommended a bootstrap method for non-normal traits.

Compared with the double hierarchical generalized linear models (using the pre-cursor to the 2011 macro- and micro-environmental model) in simulated data with strong normality (an advantage in uniformity testing), the LRT mean tests performed comparably. In the variance tests, however, the LRT_V_ performed with the highest power. In single-purpose tests, the LRT_M_ and LRT_V_ are comparable to the DHGLM_M_ and the DHGLM_V_. Further, joint tests (tests where differences in means uniformity were tested simultaneously) were never as powerful as mean tests in the presence of just mean differences, and similarly, joint tests were never as powerful as variance tests in the presence of just variance heterogeneity.

Both the DHGLM and LRT methods have been applied to real data sets. The results of the cow heard analyses indicated that the within-herd micro-environmental model had the best fit, and that selection for increased milk production increased the environmental sensitivity (Mulder et al., 2013b). In a second application to dairy traits, the environmental effect was again shown to contribute substantially to the variance (Mulder et al., 2013a). In the Cao et al. (2014) work the new method was applied to a well-understood functional variant important for Alzheimer disease; the analysis showed that closely linked SNPs with different population distributions could be detected using the method. As yet, this omnibus method has not been applied to environment or modifier detection.

Variance-detection methods have not been widely exploited in plant genetics since 2011. We found one example, in analysis of flowering time. The genetics of flowering in plants is extensively studied and this pathway was recently used for a data analysis incorporating uniformity (Shen et al., 2012). Shen et al. (2012) used the Brown-Forsythe method for variability analysis, as there were no repeated-genotype replicate measurements available in a suitable data set. They found novel, trans loci for uniformity rather than the same loci as the mean effects, which is consistent with our results. As the flowering time control pathway in *Arabidopsis thaliana* is well described, these authors were able to determine that variance control is reflected in downstream pathway components; in other words a high-variation allele for a gene early in the flowering genetic regulatory network has high-variation downstream-gene QTL. Flowering time is known to be strongly affected by a specific environmental cold treatment, vernalization. When Shen et al. (2012) examined the effect of vernalization, they found that the variance QTL are lost. This result is similar to the plasticity effect that we observed, where adding an environment resulted in reduced uniformity QTL effects as compared to the single stress environment.

In addition to improved statistical methods since 2011, and applications of those methods, there is some recent published work on combined stress environment genetics. We recently examined the genetic architecture of combined ultraviolet radiation and drought stress QTL in maize, in both the IBM94 mapping populations and a subset of the nested association NAM population (Makumburage et al., 2013). In both our examples of multiple-stress QTL mapping (plant height under drought and fertilizer limitation and several growth traits in ultraviolet radiation and drought conditions), the mean trait alleles decline less than expected from the effects estimated in the single stress cases. This is consistent with the Makumburage and Stapleton (2011) uniformity genetic architecture, where the uniformity decreased—there were no allele effects identified that increased uniformity in combined stress environments. Combined stress alleles are thus hypothesized to attenuate plasticity. Another key publication on combined stress genetics approaches the topic from a breeding perspective—Cairns et al. (2013) found that optimal genotypes under combined heat and drought were not identified as optimal in single stress environments. This provides incentive for us to better understand the mechanism of stress combination for future use in prediction and selection of test field sites.

Statistical formulations for plasticity epistasis and pleiotropy detection have been described recently (Zhou et al., 2013; Zhai et al., 2014). These models could guide model construction and fitting to combination-stress environment data for multiple traits together, instead of using qualitative map comparison as we originally did; however, the models would need to be extended to incorporate factorial treatment environment combinations to fit our data. Pathway and set detection methods (Bakir-Gungor et al., 2014; Marjoram et al., 2014) have also recently been suggested as approaches for defining more specific models to fit to datasets such as ours.

## Areas for future research

As uniformity decreases in the combination stress environment in our experiments with the maize IBM94 population, we propose that there are more modifiers involved in combination stress than in single stress responses. We suggest that a single-stress response would have a smaller network of transcription factors or physiological intermediaries, and genetic variation in those factors would be detectable in small mapping experiments. In contrast, the network of master regulators in combinations of stresses is hypothesized to balance input from different stress responses and thus be either a) a larger network with each individual transcription factor or physiological component playing a proportionally smaller, attenuating role, so that there would be no large-effect detectable allele that controls high genotype-by-environment effects in the combined-stress case, or b) have attenuating network interactions that repress QTL effects. We base our proposed mechanisms on both our results and on the more general observation that heritability decreases in stress environments. We favor the second explanation in theory, as the network of effects cannot be increased indefinitely as more environmental factors are applied, and as negative feedback/homeostasis is a defining feature of biological systems. These two hypotheses could be distinguished by increasing the power to detect small-effect QTL, either by increasing the sample size or improving the model power, or both; an increase in detection of QTL in combination stress would support the first hypothesis. In addition, we suggest that agronomically important factors such as heat and drought combinations be considered for follow-up experimental analyses; these factors are relatively difficult to manipulate and would likely require large numbers of experimental plots and careful fitting of covariates, but would provide results relevant to conditions predicted under climate change. If attenuating combined environment networks are common in elite germplasm, then simulations of breeding strategies could incorporate this constraint.

In small mapping populations such as ours there is limited statistical power for detection of combinatorial all-two-way interactions. We thus suggest that pathway or other complex biological priors be developed for follow-up analyses. Simulations and improved, easy-to use simulation methods to assist in developing priors for fitting complex causal models to the relatively small combination stress data sets that are possible for single investigators to generate would be helpful. A careful comparison of models with negative interaction latent variable structure to models with increased numbers of latent variables would also be helpful in distinguishing between our two hypotheses for combination stress genetic architecture.

Why does heritability typically decline under stress? Is it an increase in noise as the system goes out of bounds, or a recruitment of more functions? We can restate this question in the as “does Ve or Vg*g*e increase?”. The key to addressing this question is developing better ways to partition the variance and to rigorously incorporate priors such as genetic regulatory pathway architecture. We suggest further exploration of causal models using methods such as Pearl's causal graph calculus (Pearl, 2000) and comparison of semi-supervised vs. self-training analysis methods, to help untangle causality and modifiers in small, high-dimensional data sets. As a follow-up to our discussion on wide crosses for favorable alleles (Makumburage and Stapleton, 2011), we suggest that mapping of specific and general combining ability of uniformity in stress and control environments might be helpful in addressing the architecture of stress-responsive networks. Since the combining ability of uniformity can be mapped to SNPs it is not a “noise” effect, and we suggest that dynamic network models include dissection of both variances and means.

As we consider methods for complex trait and environment interaction, our choice of simulation model type becomes important. For example, are trans alleles for uniformity best thought of as nodes, as physical objects that change state such as protein that can be phosphorylated, or increased hormone concentrations, or should they be conceptualized as networks, with connections such as kinase activity or hormone movement? We suggest simulations of genetic regulatory network models to examine differences in uniformity, and systematic exploration of models using shared simulations across communities of researchers to better understand the constraints and power of different methods such as structural equation modeling and Boolean network construction. Simulations are especially useful for comparison of detection methods for precision and accuracy, and for ensuring that follow-up experiments have maximum power. Model systems for genetic architecture are also important to consider as simulations are constructed. For example, in yeast model systems that have one-step allele replacement, comprehensive simulation of knock-out effects should be part of any modeling effort. Plant model systems are especially well suited to multivariate trait data collection and analysis, and to developmental series-environment analyses, as well as to large-scale replication of genotypes by seed increase. Developmental and multi-trait factors should be incorporated into gene regulatory network models and explicitly tested for sensitivity to inform future experimental work.

### Conflict of interest statement

The authors declare that the research was conducted in the absence of any commercial or financial relationships that could be construed as a potential conflict of interest.
